# Morphological, Mechanical and Mucoadhesive Properties of Electrospun Chitosan/Phospholipid Hybrid Nanofibers

**DOI:** 10.3390/ijms19082266

**Published:** 2018-08-02

**Authors:** Ana C. Mendes, Jorge Sevilla Moreno, Muhammad Hanif, Timothy E.L. Douglas, Menglin Chen, Ioannis S. Chronakis

**Affiliations:** 1 Nano-BioScience Research Group, DTU-Food, Technical University of Denmark (DTU), B202, 2800Kgs Lyngby, Denmark; jasm@food.dtu.dk (J.S.M.); ioach@food.dtu.dk (I.S.C.); 2 Interdisciplinary Nanoscience Center (iNANO), Aarhus University, DK-8000 Aarhus C, Denmark; hanif@phys.au.dk; 3 Engineering Department, Lancaster University, Lancaster LA1 4YW, UK; t.douglas@lancaster.ac.uk; 4 Department of Engineering, Aarhus University, DK-8000 Aarhus C, Denmark; menglin@eng.au.dk

**Keywords:** chitosan, phospholipids, electrospun fibers, elastic modulus, mucoadhesion

## Abstract

This study aimed to develop hybrid electrospun chitosan–phospholipid nanofibers and investigate the effect of phospholipid (P) content and chitosans (Ch) molecular weights (Mw) and degree of acetylation (DA), on the morphological, mechanical and mucoadhesive properties of the nanofibers. Electrospun *Ch/P* nanofibers exhibited a smooth and uniform surface with average diameters ranging from 300 to 1000 nm, as observed by scanning electron microscopy (SEM). The average diameter of the nanofibers was observed to increase with the increase of the Mw and degree of deacetylation of Ch, and phospholipid content. The elastic and adhesive properties of the nanofibers were determined by atomic force microscopy, and displayed higher values for higher Mw and lower DA Ch used. The elastic modulus of electrospun *Ch/P* hybrid fibers determined for the different conditions tested was found to be in the range of 500 and 1400 MPa. Furthermore, electrospun *Ch/P* nanofibers displayed mucoadhesive properties expressed by the work of adhesion calculated after the compression of the nanofibers against a section of pig small intestine. Our results showed that the increase in phospholipid content and DA of Ch decrease the work of adhesion, while the increase of Mw resulted in slightly higher work of adhesion of the nanofibers.

## 1. Introduction

Chitosan (Ch) is a positively charged polysaccharide that consists of glucosamine and *N*-acetyl glucosamine units, obtained by partial or full deacetylation of chitin, a naturally occurring polysaccharide [1]. Due to the versatile chemistry of chitosan, which encloses different degrees of deacetylation (DD), a range of low, medium, high molecular weights (M_W_) and different distribution of the acetyl groups along the polymeric chain, chitosans can provide a range of physicochemical and biological properties [1]. Therefore, chitosan has gained a lot of attention within pharmaceutical, biomedicine and food fields due to its biocompatibility, non-toxicity, biodegradability, hemostatic activity, antibacterial, antimycotic and mucoadhesive properties [2,3,4].

Mucoadhesive materials are known for their ability to adhere to the soft mucosal surface [5] that lines the gastrointestinal, reproductive, tracheobronchial, and ocular systems [6]. The mucoadhesive properties of Ch are widely documented [7] and are known to be dependent of many mechanisms [8,9] related to the hydrogen bonding of its–OH and–NH_2_ groups to mucin, as well as electrostatic interactions [10] between the positively charged amines of chitosan and the negatively charged sialic acid residue of the mucin [9]. In addition, the molecular conformation of chitosan [9] and its hydrophobic effects can also contribute to the mucoadhesion properties [8]. 

The use of mucoadhesive polymers, such as chitosan, is required to develop carriers for transmucosal drug delivery to achieve a localized and prolonged delivery of the bioactives (approximately 12–24 h) [7]. Thus, the mucoadhesive nature of chitosan is expected to favor the extension of the residence and increase of the release time of the bioactive compound in the gastrointestinal media [10].

Mucoadhesive chitosan structures such as gels [11], particles [12] and electrospun fibers [13,14] have been reported for drug delivery applications. Fibers produced by electrospinning [15], are appealing for drug delivery applications due to their high surface area, tunable diameter and surface functionality [16,17,18,19,20] that favors diffusion of the drug, in addition to a high encapsulation efficiency.

Mucoadhesive electrospun nanofibers of chitosan (Ch) and thiolated chitosan (Ch–SH) were produced for dental caries prevention [13]. Overall, these fibers showed good mucoadhesive properties, which were significantly improved by the addition of Ch–SH, (the force required for the detachment of the Ch–SH/PVA mats from porcine buccal mucosa was two times higher than for *Ch/*PVA fibers). Furthermore, these mats were observed to release α–mangostin, which exhibited antibacterial activity on dental caries pathogens.

Mucoadhesive electrospun fibers of zein–chitosan have been prepared for the encapsulation and release of α-tocopherol (α-TOC) [14]. The addition of the acidic Ch solution to the zein containing α-TOC solution increased the polarity of zein and improved the mucoadhesion of the composite fibers containing α-TOC. Furthermore, the gastro-mucoadhesive properties were observed to be similar between simulated gastric fluid (SGF) at pH 1.2 with pepsin, and at SGF at pH 2 without pepsin. The release of α-tocopherol in release media with pepsin at pH 1.2 was observed to be triggered by the degradation of the matrix, while at pH 2 without pepsin, the release was triggered by the sudden swelling of the fiber matrix followed by the diffusion of the vitamin. 

Mucoadhesive glutamine–loaded polyethylene oxide (PEO) electrospun nanofibers were also produced [21]. Different polyelectrolytes were added to the electrospinning solution and it was observed that the work of mucoadhesion, tensile strength and elongation at break values decreased by the addition of polyelectrolytes to PEO nanofibers. PEO/sodium alginate formulations displayed the highest work of adhesion values among the formulations. Furthermore, it was observed that more than 85% of the drug was diffused from the nanofibers after 4 h in simulated saliva solution (pH 6.8, 37 °C), suggesting that glutamine loaded-electrospun-nanofibers have potential as an oromucosal drug delivery system. 

Peak force quantitative nanomechanical mapping (QNM) atomic force microscopy (AFM) mode has been used to study the mechanical properties of electrospun fibers [22]. Examples of polymers tested using this methodology include synthetic (e.g., (e.g., poly-l-lactic acid (PLLA) [22], polyvinyl alcohol (PVA) [23] and polyacrylonitrile (PAN) [24]), and also natural macromolecules (gelatin, collagen and elastin [25] and phospholipids [26]. PeakForce QNM AFM modes have been used for mapping mechanical properties simultaneously with topography, at the same spatial resolution [27].

In our recent study, hybrid electrospun chitosan/phospholipid nanofibers (*Ch/P*) were produced for drug–delivery applications [28].These fibers were stable when exposed to aqueous media for at least 7 days, due to the interactions of the amine groups of chitosan with the phospholipid counterparts. Furthermore, those fibers were non-cytotoxic and released both hydrophilic (diclofenac, vitamin B12) and hydrophobic (curcumin) drugs. 

Thus, the present study focuses on the development of electrospun chitosan/phospholipid hybrid nanofibers by using various types of Ch with different Mw and DA. Furthermore, the effect of phospholipid content and chitosans properties (Mw and DA) on the morphological, mechanical and mucoadhesive properties of chitosan/phospholipid nanofibers was investigated. 

## 2. Results and Discussion

### 2.1. Morphology of Electrospun Ch/P Nanofibers

**Figure 1:**
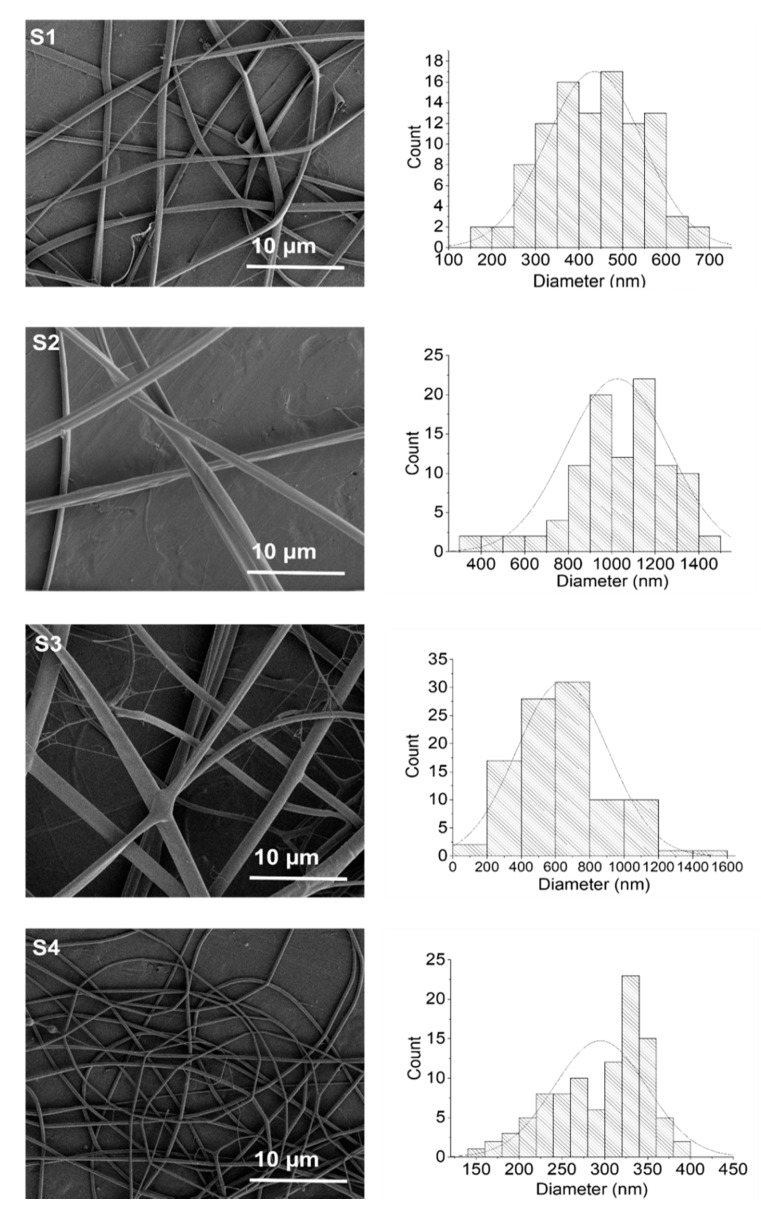
Scanning electron microscope (SEM) images of *Ch/P* fibers and respective histograms (on the right side) with the distribution of fiber diameters.

Figure 1 shows the morphology of the electrospun chitosan/phospholipid fibers. Samples with different chitosans pursuing distinct DA and Mw and the ration of chitosan: phospholipid 1:1 (*w*:*w*) were designated by S1, S3 and S4, as described in Table 6. The sample S2 used the same chitosan of S1 at the ration 1:3 (*w*:*w*) chitosan: phospholipid. Chitosan/Phospholipids nanofibers exhibited a smooth and uniform surface without any structural defects. The average diameters of the hybrid *Ch/P* S1 and S2 nanofibers was about 435 and 1030 nm, respectively. The increase of phospholipid content from sample S1 to S2 resulted in the increase in the fiber diameter by 2.4 times (*p* < 0.001). Such increase resulted from the increase of the viscosity of the solution due to chitosan–phospholipid interactions as described in a previous work [28].

Homogeneous and uniform nanofibers were also obtained with samples S3, S4 (Figure 1) with average diameters of 640 and 294 nm, respectively. In order to understand the effect of chitosans Mw in terms of fiber diameter, samples 1 and 3 with similar DA were compared. The increase of the Mw of chitosan, from sample S1 to sample S3, led to an increase in the diameter of fibers (*p* < 0.001) from 435 to 640 nm, as result of the increase of the viscosity of the solution [29].

Likewise, the role of the DA in the size diameter of the *Ch/P* fibers was also investigated by comparing S3 (DA 12%) with S4 (DA 6%). Moreover, the decrease of the degree of acetylation of the chitosan from DA 12% to DA 6% (S3 and S4), led to a decrease of the average diameter of the *Ch/P* fibers (*p* < 0.001) by more than 2 times. Most probably, the higher content of the acetyl groups resulted in less flexible polymer chains, due to the higher crystallinity and/or glass transition temperature [30].

### 2.2. Mucoadhesion Studies

#### Mucoadhesive Properties of the Chitosans

In order to predict the mucoadhesive properties of the chitosans used to produce hybrid electrospun *Ch/P* fibers, interactions of chitosans and chitosans/phospholipids with mucins were investigated through changes in size, zeta potential and turbidity of the samples, as a first step.

Turbidity measurements were used to monitor the interactions of chitosans with mucins and phospholipids and the mixtures of chitosans/phospholipids with mucins. Individual solutions of chitosans, phospholipids and mucins were used as controls. 

**Figure 2:**
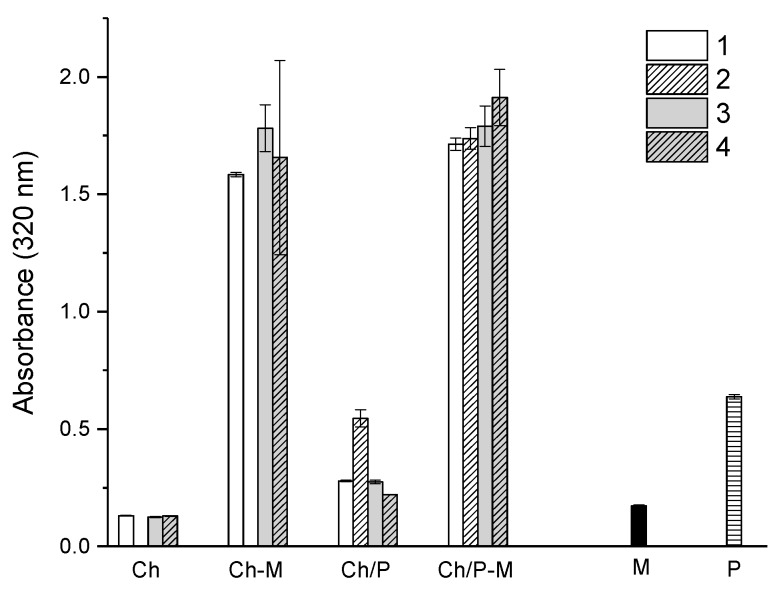
Turbidity profiles of Ch’s (samples 1, 2, 3, 4), Ch-M, *Ch/P*, *Ch/P*-M, M (Mucin) and P (phospholipids) solutions after 30 min of interactions. Presented results are average of at least three independent experiments (*n* = 3) and are presented as mean ± standard deviation.

The absorbance of the individual chitosans didn’t vary significantly from sample to sample as shown in Figure 2. The addition of mucin to the chitosans increased the measured absorbance due to the interactions between components (Figure 2). This increase is assumed to be related with the formation of chitosan-mucin nano complexes as reported elsewhere [7,31]. As illustrated in the Figure 2, all the samples exhibited a higher turbidity after the addition of mucin. Accordingly, all chitosan-based formulations interacted with mucin. However, the variations in the magnitude of the interactions, suggest differences in the mucoadhesive properties of the formulations. Ch 3 displays slightly higher absorbance (1.78) than Ch 1 (*p* < 0.05), potentially due to the slightly higher Mw which favors more interaction mucin-Ch. Ch 4 was expected to exhibit the highest absorbance due the the higher interactions of mucin–chitosan as result of lower DA. However, this was not observed. Indeed, the lower absorbance of Ch 4 comparatively to Ch 3 is not statistically significant. 

The addition of phospholipid increased the absorbance in all the Ch samples reaching the maximum of 0.53 for S2 due to the higher content of phospholipid present in the sample. S1 and S3 have similar absorbance values of 0.27 as those chitosans have similar DA, thus similar level of interactions with phospholipids would be expected. S4 has lower absorbance (0.22) comparatively to S3 (*p* < 0.02). However, upon the addition of mucin, an abrupt increase of the absorbance to 1.71, 1.74, 1.79 and 1.91 were observed for S1, S2, S3 and S4, respectively, confirming again the interactions of mucin–chitosan/phospholipids. The presence of phospholipids did not affect the interaction of mucin–chitosans and confirm the expectations in terms of binding affinity of chitosan–mucin, being the S4 the most favorable to interact with mucin due to the lowest DA, followed by S3 and S1. The combination of phospholipids with chitosan have been documented as powerful mucoadhesive carriers for the delivery of drugs [32], highlighting the potential of combining these types of molecules for mucoadhesive applications. 

The presence of higher content of phospholipid (S2) led to a lower increase in absorbance from 0.53 to 1.73 comparatively to S1 (0.279 to 1.713), suggesting that such an increase in phospholipid content from 1:1 to 1:3 (*Ch*:*P*) did not favor the increase in interactions of mucin–*Ch/P*. 

**Figure 3:**
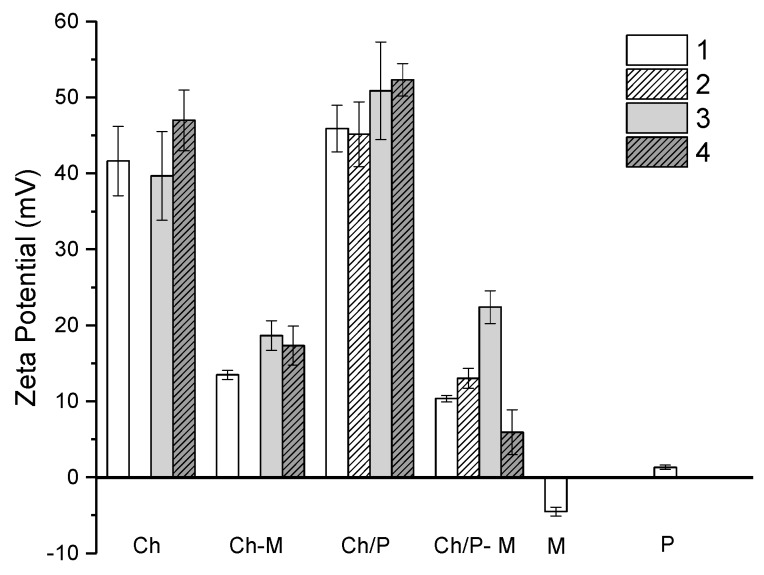
Zeta potential of Ch’s (samples 1, 2, 3, 4), Ch–M, *Ch/P*, *Ch/P*–M, M (mucin) and P (phospholipids) solutions. Presented results are average of at least three independent experiments (*n* = 3) and are presented as a mean ± standard deviation.

Zeta potential measurements are a common method to investigate the mucoadhesive properties of several biopolymers [31,33]. Mucin has negative charges, with zeta potential of approximately −5 mV, thus the positive surface charges of chitosan-based formulations are expected to interact strongly with mucin (Figure 3). Several studies have been reporting the negative zeta potential of mucins, around −7.9 mV (PGM) [31],which tends to vary with the concentration of mucin and pH. 

Chitosans, on the other hand, have a positive surface potential due to the protonation of the amino groups at pH 3.5. The zeta potential for chitosan 1, 3, 4 was found to be of 41.62 ± 4.6; 39.66 ± 5.8 and 47 ± 3.9 mV, respectively (Figure 3). The highest zeta potential observed for Ch 4 is related to the lowest DA. Chitosan 1 and 3 did not exhibit significantly different zeta potential. The addition of mucin led to the decrease of the zeta potential to 13.475 ± 0.6, 18.65 ± 1.9 and 17.3 ± 2.6 mV for samples 1, 3 and 4, respectively. This observation confirms that chitosan binds onto the mucinous aggregates, thus changing their zeta potential towards negative values. The addition of phospholipids was observed to slightly increase the zeta potential; however, these changes in zeta potential were not statistically different. Similarly to Ch–M mixtures, the addition of mucin to *Ch/P* led to the decrease of zeta potential to 10.4 ± 0.4, 13.0 ± 1.3, 22.4 ± 2.2 and 5.9 ± 2.9 mV. For most of the Ch formulations the decrease in zeta potential was more notorious in the presence of phospholipids and significant differences were found for between groups (*p* < 0.02). Therefore, this data suggests that the binding affinity to mucin follows the order S4 > S1 > S3 > S2. 

**Figure 4:**
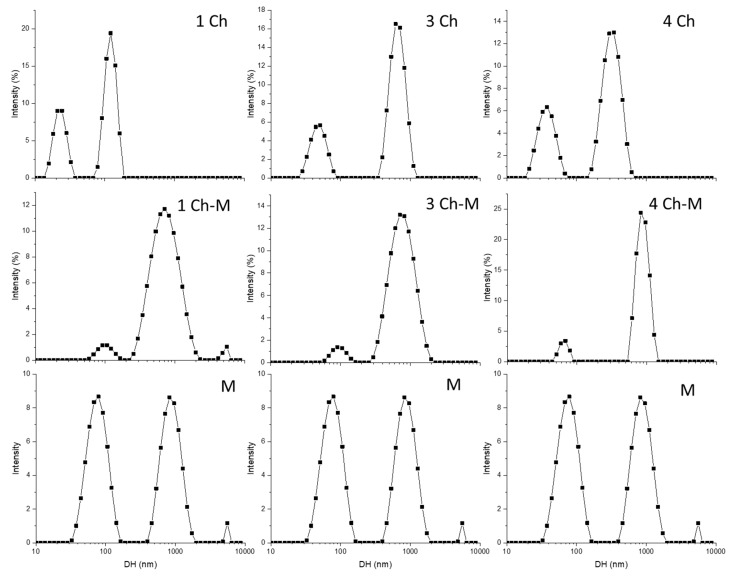
Intensity weighted particle size distributions of of Ch, Ch–M and M solutions obtained by DLS.

Figure 4 shows the intensity weighted particle size distributions of the solutions as examined by dynamic light scattering (DLS). In our study, three major populations appeared in the intensity weighted size distribution of the hydrodynamic diameter (D_H_) for mucin (M) with local maxima 87.12 ± 29 nm, 949.3 ± 272.3 nm and 5302 ± 321.4 nm (Table 1). Similar size distributions with three main populations were also found by Patil and co-workers [34]. Those authors pointed out that the multiple peaks must be associated with the presence of non-bound impurities or linkers between mucin molecules often present in commercially available mucins [34].

Chitosans on the other hand display two main populations, which vary according the Mw and DA, as can be seen in Figure 4 and Table 1.

The addition of mucin to the chitosans resulted in the increase of the hydrodynamic diameter (Table 2), as result of the binding of mucin with chitosan, as discussed previously in terms of turbidity and zeta potential (Figure 2 and Figure 3). However, this increase in D_H_ was more notorious for samples 1 and 4. 

The inclusion of phospholipid within chitosan solution was also observed to increase the D_H_ of the samples (Table 3). Furthermore, with exception for S1, only one population of particulates was observed for *Ch/P* samples. The increased size of the mixture suggested the formation of chitosan/phospholipid complexes, as a result of their interactions as reported in a previous study [28]. 

The addition of mucin to *Ch*/*P* solutions was observed to shift the main populations to 1647 ± 650, 859.99 ± 25, 888.2 ± 321.3 and 855.6 ± 291.3 nm for samples S1, S2, S3 and S4 respectively (Table 4). This increase in the hydrodynamic diameter has been previously reported as result of the binding of mucins with the biopolymeric formulation [7,31,34], confirming the mucoadhesive properties of *Ch*/*P* formulation.

### 2.3. Mucoadhesive Properties of Electrospun Ch/P Fibers

The mucoadhesive properties of *Ch*/*P* fibers were investigated. It is known that mucoadhesion of chitosan based structures is ruled by its physicochemical properties and physiological variables. The mucus in biological tissues is composed of the glycoproteins (mucins), which are negatively charged and able to interact with the positively charged chitosans, by electrostatic forces, as observed in the previous studies. Consequently, the interactions chitosan–mucins are highly dependent on the amount of sialic acid present in the mucin and on the Mw and DA of chitosan [35]. 

**Figure 5:**
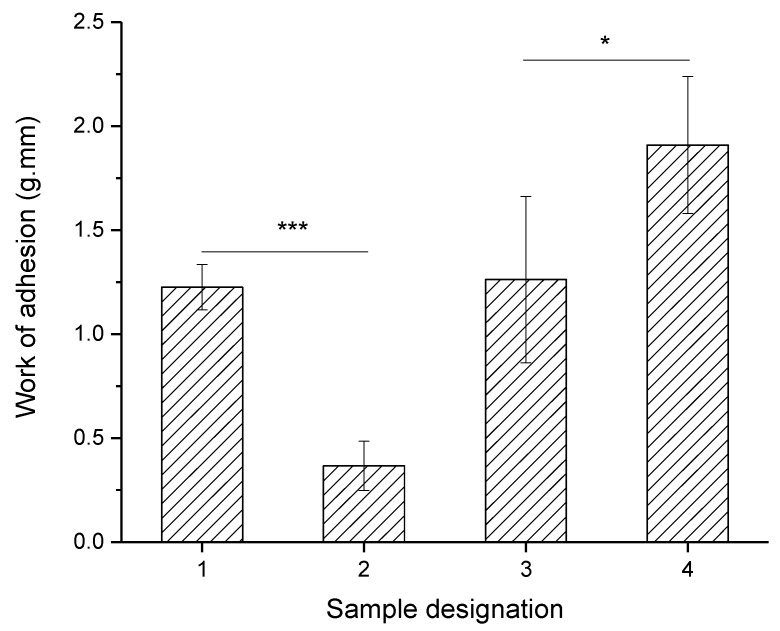
Work of adhesion of *Ch*/*P* fibers with pig small intestine, normalized with Teflon pads as a control. Results are expressed as means ± standard deviation, and are performed in triplicates, *p* < 0.05 (*) and *p* < 0.001 (***).

Mucoadhesive properties of *Ch*/*P* fibers were investigated by determining the work of adhesion resulted from the compression of the fibers against a section of pig small intestine in phosphate buffer saline (PBS) solution (pH 7.4, 37 °C). From Figure 5 it can be seen that the phospholipid content, the DA and Mw of chitosans affected the work of adhesion and mucoadhesive properties of *Ch*/*P* hybrid fibers. 

Figure 5 shows that the increase of phospholipid content by 3 times led to a significant decrease on the work of adhesion from 1.23 to 0.27 g.mm (*p* < 0.005). Mucoadhesion is known for being affected by hydrophilicity and swelling capability of a polymeric matrix [36]. Overall, for stable polymeric matrices, it has been observed that a higher flexibility and hydration rate of the matrix, will result in a larger surface area of the polymer able to interact with the mucosal area and thus favoring the mucoadhesion. However, if too much moisture is present and the degree of swelling is too high, a slippy mucilage is expected, which can be easily removed from the substrate [36]. It is known that asolectin phospholipid, which contains lecithin as a major component, shows a fast and /high absorption of water, which in turn limits its mechanical resistance as determined in weight loss studies [28] and effectiveness to adhere to the mucus layer.

The effect of Mw of the chitosan on the mucoadhesive properties of the fibers was investigated by comparing S1 with S3. *Ch*/*P* fibers containing chitosan with higher Mw (S3) was observed to display slightly higher work of adhesion (1.26 g.mm) than S1 (1.23 g.mm). Previous studies have shown that using high Mw chitosan in solution results in higher interactions between chitosan–mucin and, therefore, stronger mucoadhesion [7,37] due to the increased penetration of the Ch molecules in the mucin layer [35].

On the other hand, by comparing *Ch*/*P* fibers with similar Mw and different DA (S3 and S4), the increase of DA from 6% to 12% led to a decrease of the work of adhesion and mucoadhesive properties of the fibers from 1.91 to 1.26 g.mm (*p* < 0.5). This is related to the higher charge density within the fibers which consequently displayed more effective adhesive properties, produced using chitosan with lower DA [7,38].

Previous studies also demonstrated that the interactions of Ch–mucin in solution are significantly influenced by the degree of acetylation (DA) [7]. It is known that Ch–mucin interacts mainly electrostatically, supported by other type of interactions (e.g., hydrogen bonds and hydrophobic association). Thus the DA is expected to have a greater influence on the overall conformation of Ch and thus the nature of the resulting complexes [7].

Huang et al. found that the binding affinity and uptake capacity of chitosan molecules in solution and nanoparticles, decreased when reducing polymer Mw and degree of deacetylation [38]. Furthermore, the effect of DA on mucoadhesion was observed to be predominantly effective comparatively to the effect of Mw due to the electrostatic interactions between the positively charged chitosan microspheres and negatively charged mucus glycoproteins.

The above results suggest that sample S4 displayed higher mucoadhesive properties, highlighting the importance of the DA of chitosan to tune the mucoadhesive properties of electrospun nanofibers. Furthermore, the processing of chitosan/phospholipid with electrospinning was observed to not affect significantly the mucoadhesive properties of the chitosans tested. 

### 2.4. Mechanical, Adhesion Properties by Atomic Force Microscopy (AFM)

**Figure 6:**
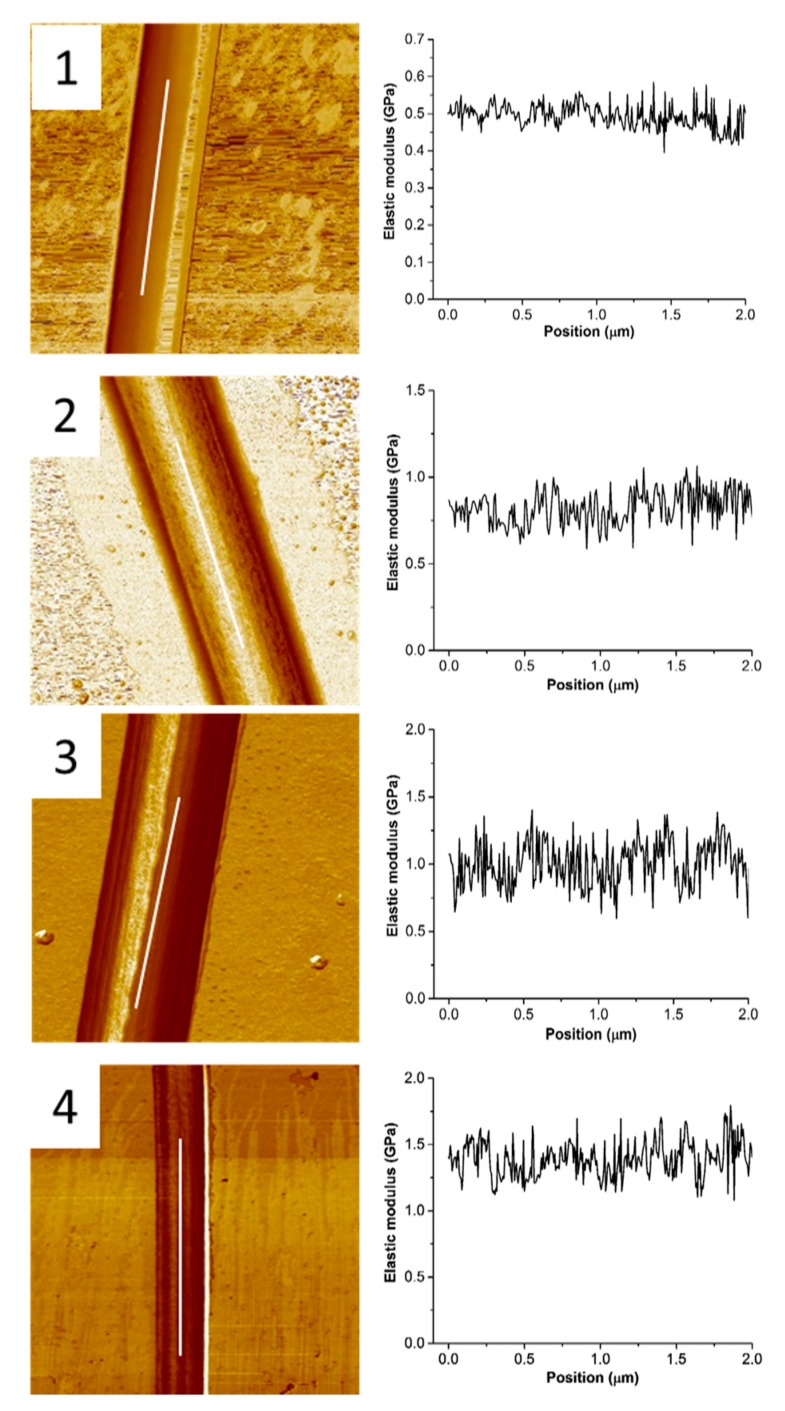
Peak force quantitative nanomechanical (PFQNM) stiffness images (left column) and the Derjaguin–Muller–Toporov (DMT) modulus (right column) of a single *Ch*/*P* nanofiber using different ratios of *Ch*/*P* (samples 1, 2) and different Mw and DA (samples 3, 4). 2 µm line profile was drawn along the major axes of nanofiber for averaging DMT values; 3 µm × 3 µm scan size.

Figure 6 shows the stiffness images and Derjaguin–Muller–Toporov (DMT) modulus obtained from peak force quantitative nanomechanical (PFQNM) examination of the single nanofibers produced with the different chitosans and phospholipid content. In order to avoid substrate contribution and instability of the edges, the DMT modulus was acquired along the central part of single nanofibers (line profile of 2 µm length). For qualitative analysis, the PFQNM was performed on nanofibers of around the same height. 

Herein, S1 exhibits a stable young modulus of approximately 500 MPa, however when increasing the content of phospholipid (S2) the modulus was observed to increase to approximately 750 MPa. Such increase in young modulus is related with the increase in the fiber diameter with the increase of the phospholipid content. Previous studies have been demonstrating that the Young’s modulus of single electrospun polymeric fiber is dependent on the diameter of the fibers [22,24,39,40]. The mechanical properties of electrospun fibers are dependent on the changes in orientation of the polymer molecules during the electrospinning process provided by the strong strain forces of the polymer jets [40]. It could be expected that having a higher content of phospholipids on the formulation would represent more opportunities for molecular rearrangements that can further change the elasticity of the *Ch*/*P* fibers. The high strain rate of the ejected polymer jets induces a molecular orientation of polymer nanofibers along the fiber axis [40].

The mechanical properties of chitosans based structures (e.g., nanofibers, hydrogels) is also known to be dependent on their molecular weight [41] and degree of acetylation [42]. Such evidence was observed when comparing S1 and S3 (similar DA and different Mw) and S3 and S4 (similar Mw and different DA). S1 (Mw 213 kDa) displayed lower elastic modulus (around 500 MPa) comparatively to S3 (Mw 293 kDa, 1000 MPa). Previous investigations demonstrated that lower molecular weight (Mw) chitosan (s) facilitates the chain rearrangements of chitosan molecules, and thus lower stiffness [41].

The decrease in DA was observed to increase the elastic modulus of electrospun *Ch*/*P* fibers from 1000 to 1500 MPa for samples with DA 12% and 6% respectively. The less acetylated chitosan is expected to have a higher amount of positive charges able to interact with negative groups of the phospholipids, as reported [28], and thus providing higher elasticity. 

Schauer and co-authors determined the Young’s modulus of the as-spun and cross-linked chitosan fibers (medium Mw ranging from 190 kDa to 310 kDa), to be of 154.9 MPa [30]. The average elastic modulus of electrospun asolectin fibers determined in a previous study was about 17.3 MPa [26]. Therefore, it is suggested that *Ch*/*P* displayed higher elasticity than the pure chitosan or phospholipids electrospun nanofibers, due to the intermolecular bonding between chitosan–phospholipids hybrid nanofibers. The elastic modulus of chitosan structures can be controlled with the presence of crosslinkers and by the extension of crosslinking, or by the presence of counter-ionic specimens (e.g., ions of opposite charge, such as the ones existent in phospholipids) [41].

The elastic modulus determined for electrospun nanofibers made of synthetic polymers has been demonstrated to be dependent on the diameter size of the fiber. Electrospun nanofibers made of poly-L-lactic acid (PLLA), polyvinyl alcohol (PVA) and polyacrylonitrile (PAN) displayed an elastic modulus ranging from 0.5 to 0.9 GPa [22], 4 to 13 GPa [23] and 5.72 to 26.55 GPa [24], respectively. In addition, electrospun nanofibers made of natural materials, exhibited a lower elastic modulus (tensile moduli), compared to synthetic polymers. Examples include proteins like gelatin, collagen and elastin, with an elastic modulus of approximately 426, 262 and 184 MPa, respectively [25]. Thus, electrospun *Ch*/*P* fibers displayed reasonable modulus of elasticity above the average for electrospun fibers made of natural polymers.

Adhesion of the fibers to the probe was also analyzed by AFM (Table 5). The increase in phospholipid content seems to decrease the adhesion to the hydrophilic silicon probe from 13 to 4 nN. In addition, the use of Ch with increased Mw (S3) was observed to result in a slightly increased adhesion of 17 nN, comparatively to S1 (13 nN). Those trends are in agreement with the mucoadhesion studies (Figure 6). Furthermore, an increase in adhesion from 17 (S3) to 19 nN (S4) was observed with the increase in DA due to the increase of amino groups, which are hydrophilic, following a similar trend observed in the mucoadhesion analyses. 

S2 displays higher dissipation (1200 eV) than S1 (800 eV) as observed in Table 5. It is assumed that the increase in phospholipid content led to a strong adhesion hysteresis, due to the indentation-induced contact between the AFM probe and the hydrophilic polymeric core. Lecithin is one of the main components of asolectin with a relatively hydrophilic head group [43,44]. Thus one would expect that increasing the phospholipid content on electrospun *Ch*/*P* fibers might lead into an association mechanism of the AFM probe with the hydrophilic inner side of the *Ch*/*P* fibers, such as hydrogen bonding formation, which further contributes to high adhesion hysteresis. Samples with the same content of phospholipid (S1, S3 and S4) show similar mean dissipation values. 

## 3. Materials and Methods

### 3.1. Materials

Chitosan samples were obtained either from Gillette Chitosan (product 112) (Veraval, India) or from Heppe Medical Chitosan (Halle (Saale), Germany) and used as received. The properties of the chitosans used in this study are described in Table 5. Asolectin from soybean (25–33% of lecithin, cephalin and phosphatidylinositol, 24% saturated fatty acids, 14% mono-unsaturated and 62% poly-unsaturated fatty acids) and all other reagents were purchased from Sigma-Aldrich (Steinheim, Germany), and used as received.

### 3.2. Electrospinning 

Chitosan/phospholipid solutions were prepared as described by Mendes et al. [28]. Chitosans were dissolved in a mixture of trifluoroacetic acid/dichloromethane *TFA*/*DCM* (70:30) at a concentration of 2% *w*/*v*. Asolectin was then added to chitosan solutions at different ratios, as mentioned in Table 6. The mixture was stirred for 1 h. The processing of *Ch*/*P* solutions by electrospinning was conducted at room temperature by applying a voltage of 25 kV (Gamma High Voltage Research, Ormond Beach, FL, USA) to the polymer solution at a feed rate of 0.02 mL/min (syringe pump from New Era Pump Systems, Farmingdale, NY USA) using a 24 G needle (Proto Advantage, Ancaster, ON, Canada). *Ch*/*P* fibers were collected on a stainless steel plate covered with aluminum foil placed 10 cm from the needle tip. For the PeakForce (Quantitative NanoMechanical) QNM analysis, the electrospun sample was collected on a glass substrate.

### 3.3. Morphology

*Ch*/*P* fibers were mounted on aluminum stubs and sputter-coated (Leica Coater ACE 200 Brønshøj, Denmark) with a layer of 6 nm of gold prior to visualization in a scanning electron microscope (SEM) (Quanta FEG 3D SEM, FEI, Hillsboro, OR, USA). The average fiber diameters and diameter distributions were determined by measuring 100 fibers using the image visualization software Image–J (National Institutes of Health, Bethesda, MD, USA) 

### 3.4. Mucoadhesive Properties of Ch/P Solutions and Fibers

#### 3.4.1. Chitosans and Chitosan/Phospholipid–Mucin Interactions

Mucin from porcine stomach (type II, Sigma-Aldrich) was purified according to Menchicchi et al., 2014 [7].

For turbidity, zeta potential and DLS measurements, solutions of mucin, chitosans and chitosans/phospholipids were prepared using trifluoroacetic acid (TFA) 0.1% *v*/*v* as a solvent. Chitosans were dissolved at concentrations of 8 mg/ mL, while mucins were dissolved at the concentration of 16 mg/ mL. Phospholipids (asolectin) was then added to chitosan solutions at different ratios, 1:1 (*w:w*) to the samples 1, 3, 4 and at the ration *1:3* (*w*:*w*) to the sample 2. The mixtures were stirred overnight under continuous magnetic stirring. Each solution was filtered using pyrogen free, 0.45-mm disposable membrane filter (Schleicher and Schuell Bioscience, Dassel, Germany). 

#### 3.4.2. Turbidity Measurements

Turbidity measurements were performed after mixing chitosans (Ch), chitosans with mucin (Ch-M), chitosans with phospholipids (*Ch*/*P*) and chitosans/phospholipids with mucins (*Ch*/*P*–M) to provide information about potential interactions between the mixtures comparatively to the individual components used as controls. Those mixtures were carried out in 96 well plate with a final volume of 200 µL in each well. Turbidity profiles of Ch, Ch-M, *Ch*/*P*, *Ch*/*P*-M solutions were assessed by the changes in absorbance at the wavelength of 320 nm after 30 min of incubation of the samples at room temperature in an EnSpire Multimode Plate Reader (PerkinElmer, Skovlunde, Denmark). These studies were conducted in triplicate for each condition (*n* = 3).

#### 3.4.3. Size and Zeta Potential

To investigate zeta potential and size of the chitosan mucin complexes formed by the mixtures DLS was used. Therefore, the different samples were measured by collecting 50 µL of the sample from the 96-well plate and dissolving in 950 µL of ultrapure water. The solutions were homogenized by gently pipetting prior measurements. Both zeta potential and size analysis were performed using a Malvern Zetasizer NanoZS (Malvern Instruments, Worcestershire, UK). Zeta potential was measured by mixed laser Doppler electrophoresis and phase analysis light scattering. The size distribution of the Ch–mucin complexes was determined by DLS with non-invasive back scattering at an angle of 173°.

#### 3.4.4. Mucoadhesive Properties of *Ch*/*P* Fibers

The evaluation of the mucoadhesive properties of the *Ch*/*P* fibers was carried out by a texture analyser (Stable Micro Systems, Surrey, UK) with a 50 N load cell equipped with a mucoadhesive test ring. 

Fresh pig mucosal tissue was rinsed with phosphate-buffered saline (PBS) at pH 6.8, cut into pieces of 2.5 × 2.5 cm and placed in the mucoadhesion test ring. The mucoadhesion test ring/pig mucosal tissue were equilibrated at 37 °C in PBS, pH 6.8. *Ch*/*P* fibers collected on aluminium foil were pasted on a probe (1 cm diameter) using carbon pads prior the test. The assay consisted of placing fibers in contact with the mucosal tissue with 20 g of force for 1 min, and then withdrawn. To calculate the work of adhesion necessary to separate *Ch*/*P* fibers from the mucosal tissue the area under the curve of force versus the distance obtained from the software (Exponent, Stable Micro Systems, Surrey, UK) of the texture analyzer was determined. Teflon films of 1 cm in diameter were used as control samples. All the samples were tested three times. 

### 3.5. Atomic Force Microscopy (AFM) Nanomechanical Mapping

A PFQNM study of the samples was performed using a Bruker Dimension Icon microscope. This method allows the mapping of the local elastic properties with lateral nanometer resolution and consists on the acquisition of force curves recorded at each pixel of the topographic image. The peak force-tapping mode precisely controls imaging force, keeping indentations small to deliver non-destructive and high-resolution imaging. 

The cantilever’s physical parameters are the key to measure the tip-sample interaction forces quantitatively. Then, provided that parameters such as cantilever spring constant and tip geometry are calibrated, the quantification of the nanomechanical properties is possible. In this study, a commercially available cantilever from Bruker (TAP150A) with a resonant frequency of 135–154 kHz was used. The nominal probe spring constant was 5 N/m and the actual spring constant of the cantilever was measured by thermal tune method (approx. 3.5 N/m). The estimated tip radius (35 nm) measured from NanoScope Analysis software was larger than the nominal tip radius (8 nm) provided by the supplier. 

To determine the reduced Young’s modulus, the retract curve was fitted into the DMT model [45]:

(1) $$F-F_{adh}=\frac{4}{3}\;E^{*\sqrt{R{\left(d-d_{0}\right)}^{3}}}$$

where *F − F_adh_* is the force on the cantilever relative to the adhesion force, R is the tip radius, *d* − *d*_0_ is the sample deformation, and E* is the reduced Young’s modulus. 

The measurements were performed at room temperature and ambient conditions. Samples were imaged by using AFM operating in PFQNM in the air at a scan rate of 0.4 Hz. Each set of NanoMechanical measurements on a sample corresponds to 512 × 512 force-distance curves taken over electrospun *Ch*/*P* fibers. Data analysis was performed using NanoScope Analysis ver. 1.50 (Santa Barbara, CA, USA), dedicated software provided by the microscope producer. QNM properties were processed through SPIP 6.4.4 (Image Metrology A/S, Hørsholm, Denmark). 

### 3.6. Data Analysis

Statistical significance between samples was determined by using Student’s *t*–test function in Excel software. One-tailed unpaired *t*-test with 95% confidence interval was considered statistical significant if *p* < 0.05 (*), *p* < 0.01 (**) and *p* < 0.001 (***).

## 4. Conclusions

Uniform and homogeneous hybrid electrospun chitosan/phospholipid nanofibers can be produced using chitosan (s) with different Mw and DA. The fibers’ diameter, the mucoadhesive and mechanical properties were observed to be dependent on the phospholipid content and chitosans Mw and DA. The processing of chitosan/phospholipid with electrospinning was observed to do not affect significantly the mucoadhesive properties of the chitosans tested in solution and the presence of phospholipids 1:1 *w*:*w* (chitosan:phospholipids) was observed to favor interaction chitosan–mucin in solution. The increase in the phospholipid content by 3 times led to the increase of the fiber diameter by 2.4 times. Moreover, the increase in P content was observed to decrease the interactions with mucins and mucosal pig intestine, as observed in terms of turbidity, zeta potential and DLS analysis and by the decrease of work of adhesion, respectively. Similarly, the decrease of the adhesion determined by AFM was also observed to decrease, contrasting with elastic modulus and dissipation that increased with the increase of phospholipid content, as result of molecular rearrangements of phospholipids during the electrospinning process. 

On the other hand, the increase in Mw of Ch was observed to increase the fiber diameter, mucoadhesivness to pig tissue, elastic modulus and adhesion to the AFM probe. The decrease in DA led to a decrease in average *Ch*/*P* fiber diameter and to an increased mucoadhesivness to pig tissue, elastic modulus and adhesion to the AFM probe, due to the more available charges able to better interact with the adhesive matrices. The elastic modulus of electrospun *Ch*/*P* hybrid fibers determined for the different conditions tested was found to be in the range of 500 and 1400 MPa, depending mostly on the Mw and DA of chitosan used. Those values are above the average for electrospun biopolymeric fibers reported in the literature. 

Overall, it was found that electrospun *Ch*/*P* nanofibers displayed desired mechanical and mucoadhesive properties, which can be controlled by the type of chitosan used and phospholipid content, confirming their potential to be used in drug delivery applications. 

## Figures and Tables

**Table 1:** Values of the hydrodynamic diameter (D_H_) and the relative amount (RA (%)) as obtained by dynamic light scattering (DLS) for chitosans Ch, mucin (M) and phospholipids (P).

M		P		1 Ch		3 Ch		4 Ch	
D_H_ (nm)	RA%	D_H_ (nm)	RA%	D_H_ (nm)	RA%	D_H_ (nm)	RA%	D_H_ (nm)	RA%
87.12 ± 29	45.1	382.3 ± 33	100	193.6 ± 42.35	59.5	292.9 ± 35.27	81.4	329.1 ± 91.28	68.6
949.3 ± 272.3	44.7	-	-	32.48 ± 10.97	40.5	23.73 ± 2.9	18.6	38.20 ± 10.12	31.4
5302 ± 321.4	3.7	-	-	-	-	-	-	-	-

**Table 2:** Values of the hydrodynamic diameter (D_H_) and the relative amount (RA (%)) as obtained by DLS for chitosans–Mucin (M).

1 Ch-M		3 Ch-M		4 Ch-M	
D_H_ (nm)	RA%	D_H_ (nm)	RA%	D_H_ (nm)	RA%
872.5 ± 418.2	93.9	812.5 ± 312.8	94.2	832.6 ± 151.2	59.5
104.2 ± 24.5	6.1	96.79 ± 21.6	5.8	65.54 ± 9.1	40.5

**Table 3:** Values of the hydrodynamic diameter (D_H_) and the relative amount (RA (%)) as obtained by DLS for chitosans/Phospholipids (*Ch*/*P*).

1 *Ch*/*P*		2 *Ch*/*P*		3 *Ch*/*P*		4 *Ch*/*P*	
D_H_ (nm)	RA%	D_H_ (nm)	RA%	D_H_ (nm)	RA%	D_H_ (nm)	RA%
529.2 ± 112	89.3	625.0 ± 112.9	100	624.8 ± 132	100	648.9 ± 137.5	100
84.73 ± 11.7	10.7	-	-	-	-	-	-

**Table 4:** Values of the hydrodynamic diameter D_H_ and the relative amount (RA (%)) as obtained by DLS for chitosans/Phospholipids–Mucin (*Ch*/*P*)–M).

1 *Ch*/*P*–M		2 *Ch*/*P*–M		3 *Ch*/*P*–M		4 *Ch*/*P*–M	
D_H_ (nm)	RA%	D_H_ (nm)	RA%	D_H_ (nm)	RA%	D_H_ (nm)	RA%
1647 ± 650	88.8	859.99 ± 25	93.7	888.2 ± 321.3	93.8	855.6 ± 291.3	93.5
290.2 ± 11.7	11.2	171.9 ± 34.2	6.3	115.8 ± 22.4	6.2	97.68 ± 32.43	6.5

**Table 5:** Adhesion and dissipation of electrospun *Ch*/*P* fibers.

Samples Designation	Chitosan Mw/kDa	Chitosan DA/%	Chitosan: Phospholipids/*w*:*w*	Mean Dissipation/eV	Adhesion/nN
S1	211	13	1:1	800	13
S2	211	13	1:3	1200	4
S3	287	12	1:1	700	17
S4	276	6	1:1	600–750	19

**Table 6:** Samples used in this study.

Samples Designation	Chitosan Reference	Chitosan Mw/kDa	Chitosan DA/% ^a^	Chitosan: Phospholipid/*w*:*w*
S1	Chitosan 112	211	13	1:1
S2	Chitosan 112	211	13	1:3
S3	85/2500	287	12	1:1
S4	95/3000	276	6	1:1

^a^ determined by ^1^H nuclear magnetic resonance (NMR).
